# Ultrasound-assisted extraction of phytochemicals from green coconut shell: Optimization by integrated artificial neural network and particle swarm technique

**DOI:** 10.1016/j.heliyon.2023.e22438

**Published:** 2023-11-19

**Authors:** Poornima Singh, Vinay Kumar Pandey, Sourav Chakraborty, Kshirod Kumar Dash, Rahul Singh, Ayaz Mukarram shaikh, Kovács Béla

**Affiliations:** a Department of Bioengineering, Integral University, Lucknow, Uttar Pradesh, India; b Division of Research & Innovation (DRI), School of Applied & Life Sciences, Uttaranchal University, Dehradun, Uttarakhand, India.; c Department of Food Processing Technology, Ghani Khan Choudhury Institute of Engineering and Technology (GKCIET), Malda, West Bengal, 732141, India; d Faculty of Agriculture, Food Science and Environmental Management Institute of Food Science, University of Debrecen, Debrecen, 4032, Hungary

**Keywords:** Green coconut shell, Phytochemicals, Ultrasound-assisted extraction, Particle swarm optimization, Artificial neural network

## Abstract

This study employs artificial neural network (ANN) and particle swarm optimization (PSO) to maximize antioxidant and antimicrobial activity from green coconut shells. Phytochemical analysis was carried out on the extract obtained from ultrasound-assisted extraction performed at different combinations of time (10, 20, and 30 min), temperature (30, 35, and 40 °C), and the ratio of solid-solvent (1:10, 1:20, and 1:30 g/ml). The presence of these bioactive compounds exhibits antimicrobial and antioxidant activities. Quantitative analysis showed that the total phenolic compounds ranged from 7.08 to 33.46 mg GAE/g, flavonoids ranged from 2.09 to 28.46 mg QE/g, tannins ranged from 70.5 to 141.09 mg TAE/g, and antioxidant activity of 49.98–66.1 %. The FTIR analysis detected the presence of C=O, O–H, and C–H bonds. The optimized condition of ultrasound-assisted extraction (UAE) was compared with the optimized condition of the microwave. The result of ultrasound-assisted extraction was observed to be better than microwave-assisted extraction.

## Highlights

• Coconut shell contains phytochemicals such as Polyphenols, tannins, and phytosterols.
• Coconut shells include flavonoids and phenolic acids.
• Lignans, phytoestrogens in coconut shells, could regulate hormones.
• Coconut tannins are antibacterial, antioxidant, and anti-inflammatory in nature.

## Graphical abstract

**Figure:**
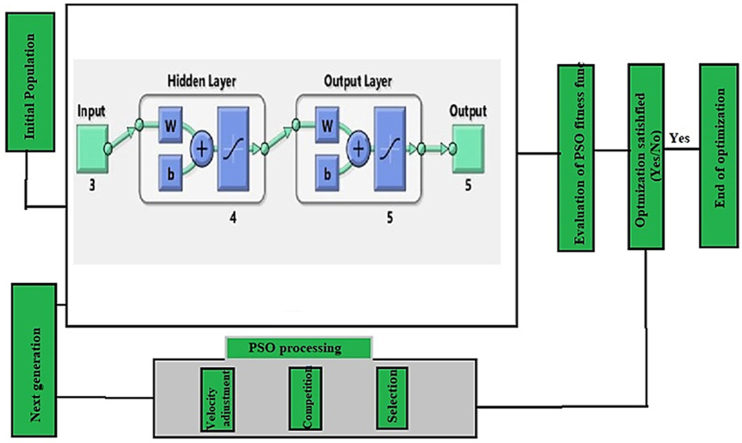

## 1 Introduction

Processing plant-based foods can produce by-products that are high in bioactive substances, such as phenolic compounds, which can have a variety of physiological effects, including those that are anti-allergenic, anti-microbial, cardioprotective, anti-inflammatory, antioxidant, and vasodilator [1,2]; Hung, 2016; [3]. The positive benefits of phenolic compounds on public health have been linked to their antioxidant capacity. Cardiovascular disease, diabetes, cancer, and many other chronic diseases along with age-related diseases, are thought to be influenced by excessive oxidative stress. Consuming antioxidants is thought of as a preventative measure to lessen the bad consequences brought on by oxidative stress [4]; Li et al.*,* 2015). Oxidative stress is a risk factor that can be brought on by numerous risk factors, including smoking, radiation, and environmental contaminants. By preventing the onset or growth of an oxidative chain reaction, antioxidants can postpone or prevent the oxidation of biomolecules (such as DNA, lipids, and protein) in tissues and cells [5]. The natural antioxidants in food may also be able to reduce hydro-peroxides into stable derivatives of hydroxyl, inactivation metal catalysts using chelating agents, and work in concert with other reducing chemicals additionally with their ability to scavenge free radicals found in the body [6]. The natural antioxidants are mostly present in cereals, spices, fruits, vegetables, and herbs and many of them have shown potent anti-inflammatory and free radical scavenging properties [1,2]; Hung, 2016; [3]. The efficient extraction of radicals from plant sources is necessary due to the growing use of these compounds in medicines and food processing.

In India, the coconut is among the five *Devavarikshas* (God's trees) and is considered a very useful tree in the world. The uses of coconut can be judged by the Indonesian saying “There are many uses of coconut as there are days in the year”. *Cocos nucifera* L. member of the Arecaceae family, a tree of the palm family, and the edible portion is coconut fruit. Typically, it is found on sandy shorelines of tropic regions [7]. The coconut has been a great source of versatility. Coconut probably originated from Indo-Malaya and spread in tropical regions of the world [7]. In India, the largest producers of coconut are the states of Kerala, Karnataka, and Tamil Nadu. The annual production of coconut fruit is 61.4 million metric tons with worldwide cultivation of 12.3 million hectares (Statista, 2020). Coconut fruit is surrounded by a hard shell and comes under the subcategory of Drupes. The fruit composes of trisection: the endocarp, mesocarp, and exocarp. Both exocarp and mesocarp make up coconut husk while green coconut shell is made by endocarp. Coconut flesh contains a high amount of fat and can be eaten in dried, fresh, and processed forms. The nut contains a liquid, known as coconut water. A hard shell encloses an embryo that is rich in endosperm and comprises both meat and liquid [7]. The green coconut shell is the waste left after coconut water and cream is removed which accounts for almost 85 % of total weight and is rich in cellulose, phytochemicals, antimicrobial and antioxidant properties. Apart from edible components, approximately 60–65 % of coconut waste is generated in the form of husk and shell [8]. Coconut shell is one of the wastes and environmental concerns in India as well as in coconut-producing countries. The green coconut shell is rich in phytochemicals such as flavonoids, reducing sugars, carbohydrates, saponins, alkaloids, tannins, glycosides, phenols, and terpenoids [[9], [10], [11]].

Currently, only a few sectors and agriculture industries use coconut fiber waste from coconut shells. It is novel to use the shells of coconut as a reservoir of chemicals, particularly phenolic compounds. The old Soxhlet procedure is losing popularity in place of ultrasound extraction which is being used in the food and pharmaceutical industries. UAE has emerged as a feasible alternative to conventional extraction techniques due to its high efficiency, minimum water usage, and low energy need [12]. Additionally, UAE is a tried-and-true method for utilizing plant material, particularly for extracting molecules with low molecular weight and bioactive plant components. As a result of the cavitation bubbles collapsing, cell walls are ruptured, particle size is reduced, and cell content mass transfer to the solvent is improved, all of which contribute to the extraction process being improved by ultrasound [[12], [13], [14]]. To extract the most antioxidants and antimicrobial agents from plant materials, it needs to optimize the extraction conditions. RSM is a useful tool for improving the process parameters because it is a statistical and mathematical technique. Utilizing RSM may make it possible to reduce the number of trials in the experiment and quantify how different parameters interact with one another. Further, an artificial neural network (ANN) is a black-box modeling approach, applied for the mapping of independent and dependent variables. In RSM-based optimization, ANN is frequently applied for the modeling of factors and responses along with other available nonlinear models. This makes the prediction more robust and accurate for the prediction of responses by the optimization algorithm. Many researchers have implemented, ANN and RSM based integrated approaches for the modeling and optimization of various extraction conditions of biological materials [[15], [16], [17], [18]]. Based on this the objective of this study is to maximize the extraction of antioxidants and antimicrobial activities from green coconut shells and study the impacts of the extraction process parameters (methanol, ultrasonication temperature, time, and the ratio of solvent-solid) by applying an integrated approach of artificial neural network (ANN) and particle swarm optimization (PSO). Additionally, MAE and UAE were used to compare the phytochemicals, antimicrobial, and antioxidant activity of green coconut shell extracts. Additionally, using FT-IR, the polyphenolic components of extracts produced under optimal circumstances were measured.

## 2 Material and methods

### 2.1 Raw material

The green coconut shell (sample) was gathered from the local shops of Lucknow, Uttar Pradesh (India). It was cut into small fragments and dried using a hot air oven for 24 h at 50 °C. A grinder was used to reduce the sample size followed by sieving to pass through a 150-μm sieve and stored in a sterilized airtight container. The standard of phytochemicals extraction (Folin –Ciocalteu reagent, Na_2_CO_3_, Gallic acid, Folin- Denis reagent, Tannin Acid, Aluminium Chloride, and Quercetin) and Potassium Acetate were purchased from Himedia. The methanol of analytical grade was used for extraction. The DPPH was purchased from GX chemicals for antioxidant activity. The standard bacterial strains *E. coli* and *S. aureus* were obtained from IIRC, Integral University, Lucknow, to study the antimicrobial effect.

### 2.2 Methods of extraction

#### 2.2.1 Ultrasound-assisted extraction (UAE)

The extract from green coconut shell powder was recovered using an ultrasound probe sonicator. In a conical flask of 500 ml containing methanol, 10 g of sample were transferred (ratio of 1:10, 1:20, and 1:30). The extraction was performed at various combinations of time-temperature. The extraction time was 10, 20, and 30 min. The temperature used was 30, 35, and 40 °C. After extraction, the mixture was filtrated using Whatman filter paper, which was dried to a constant weight at a temperature of 50 °C [14,19].

#### 2.2.2 Extraction using microwave

The extract from green coconut shell powder was recovered using a microwave. The green coconut shell powder will be mixed with solvent methanol in a Solvent Volume of 1:50. The green coconut shell and solvent processing mix were exposed to MAE processing value of power was 300 Watt and time was 2 min combination for the extraction of phytochemicals [10]. After extraction, the extract was filtered and dried at a constant weight same as mentioned above.

#### 2.2.3 Experimental design and optimization

The phytochemical extraction from the green coconut shell was conducted using the Box Behnken design (BBD). Three significant independent variables were sonication time (X_st_), temperature (X_T_), and solid-solvent ratio (X_r_). Table 1A lists the ranges of the independent variables along with the relevant codes and levels. A total of 18 experiments were conducted based on the Box Behnken-based experimental design combinations by considering five responses namely Yield (%), Phenols (GAE/g), Flavonoids, (QE/g), Tannins (TAE/g) and Antioxidants (%) for this extraction process from the green coconut shell.

**Table 1A:** Coded levels for independent variables in UAE.

Independent variables	Symbol	−1	0	1
**Treatment time (min)**	A	10	20	30
**Ultrasound temperature (**°C)	B	30	35	40
**Solvent volume (g/ml)**	C	10	20	30

### 2.3 Artificial neural network (ANN) modeling

The development of the ANN model is based on the natural neuron. Information processing in ANN models results from interactions between several simulated neurons. The artificial neural network's key elements include the input layer, hidden layer, summation function, threshold function, and output layer. By linking several processing components with varied weights, artificial neural networks can be created. Each layer is connected to the layer below by interconnection strengths or weights. In a neural network, W_ih_ are the hidden (L_H_) and input (L_I_) layer interconnection weights, whereas Who are the hidden (L_H_) and output (L_O_) layer interconnection weights. In order to minimize mistakes, weights are adjusted through back propagation. It is accomplished by adjusting initial estimated weight values (i.e. W_ih_ and W_ho_) acquired between projected and known outputs throughout the training phase. Different neurons make up the input (L_I_) and output (L_H_) layers, which represent the process's input and output, respectively [[20], [21], [22], [23]].

HL_im_, a linear function of the inputs (X_i_) and weights (W_im_) on these connections, stands in for the input to the hidden layer for the mth neuron. The mathematical expression for HL_im_ is presented in Eqn. (1).

(1) $${HL}_{im}=\sum_{i}X_{i}W_{im}$$

Bias (B_m_) values are additionally added as inputs to the hidden (B_h_) and output (B_o_) layers when the ANN architecture is processed. The neural network calculation makes use of a transfer function called *tansig*. Hence, Equation (2) provides the mathematical expression for HL_om_.

(2) $${HL}_{om}=\frac{2}{1+e^{-2\left({\text{HL}}_{\text{im}}+B_{h}\right)}}-1$$

Equation (3) was used to calculate O_m_, the output layer's ultimate result.

(3) $$O_{m}=W_{ho}{HL}_{om}+B_{o}$$

The experimental data for this investigation were split into three groups: training, testing, and validation. Programming was done in MATLAB R2015a to create ANN models. The investigation used a feed-forward back-propagation neural network. For the current investigation, ultrasound assisted extraction from coconut shell powder, five neurons viz. Yield (%), Phenols (GAE/g), Flavonoids, (QE/g), Tannins (TAE/g) and Antioxidants (%) made up the output layer and three neurons viz. sonication time, temperature, and solid-solvent ratio made up the input layer of the network. While the neuron in the hidden layer was changed after each run to get the highest coefficient of determination (R2) and lowest mean square error (MSE) values.

### 2.4 Optimization by using particle swarm optimization (PSO)

For the purpose of optimizing the constructed models of the ultrasound-assisted extraction from coconut shell powder, the standard particle swarm optimization (SPSO) method was employed. The SPSO algorithm used the chosen model as its objective function. In MATLAB R2019a, the SPSO algorithm was programmed. SPSO is a sophisticated population-based evolutionary optimization technique that replicates the flocking behavior of birds. This optimization technique can successfully and efficiently identify solutions among a certain population because it is based on collaboration and competition. According to previous observations of other particles, each particle travels in a M-dimensional space Z, and its location and velocity are described as $\vec{u_{i}}$ = (u_i1_, …,u_im_, …u_iM_) and $\vec{x_{i}}$ = (x_i1_, …,x_im_, …,x_iM_) respectively.

The search process of the particles takes place according to Equations (4), (5):

(4) $$u_{im}={wu}_{im}+c_{1}\times rand\left(\;\right)\times \left(P_{im}-x_{im}\right)+c_{2}\times rand\left(\;\right)\times \left(p_{gm}-x_{im}\right)$$

(5) $$x_{im}=x_{im}+v_{im}$$

Here, x_im_ denotes the particle's location in the mth dimension, vim denotes its velocity in the mth dimension, c_1_ and c_2_ denote acceleration constants, and rand () denotes a random number between 0 and 1. The vector pi represents the position of the particle I with the best fitness value, whereas the vector p_g_ represents the position of the best particle. w stands for the inertia weight used to balance the effectiveness of global and local search.

### 2.5 Analysis of phytochemicals

#### 2.5.1 Extraction yield

The quantity of extract obtained was compared with the initial sample amount known as the extraction yield (%), which measures how effectively the solvent extracts particular components from the original material [24]. For each approach that was examined. After drying, the dry extract was recovered; it was weighed to determine the extraction yield. The yield of the obtained extract was estimated using Equation (6).

(6) $$\text{Yield}\left(\%\right)=\frac{\text{Weight}\;\text{of}\text{crude}\;\text{extract}\;\text{recovered}}{\text{Dry}\;\text{sample}\;\text{weight}}X\;100$$

#### 2.5.2 Estimation of phenolic content

A 200 μL sample was mixed with 1.5 mL of diluted FC (Folin-Ciocalteu) reagent (1:10, v/v) and kept in incubation for 5 min at room temperature [25]. After that, 1.5 mL of 0.566 M Na_2_CO_3_ was mixed with the solution. The absorbance of the solution was measured by spectrophotometer at 725 nm, after 90 min of incubation. The same analytical process as samples was used to create the standard Gallic acid range of 0–125 mg/ml. The result was represented in milligrams of GAE per gram of material [10].

#### 2.5.3 Estimation of Tannin content

The tannin estimation was done using [10] method. A volumetric flask (50 ml) containing distilled water (20 ml), Folin-Denis reagent (2.5 ml), and 17 % Na_2_CO_3_ (10 ml) was pipette with one ml of sample extract. The mixture was properly mixed and made 50 mL using distilled water and kept for 20 min or until a bluish-green color emerged. The identical procedures used with the sample above were used with tannic acid solutions in the 0–500 ppm range. After the bluish-green hue had fully formed, the absorbance of the tannic acid (reference solutions), as well as the samples were measured using a spectrophotometer at 760 nm. Tannin concentration was determined using TASC (tannic acid standard curve), which was represented in milligrams of Tannic acid equivalence (TAE) per 100 g of dried material.

#### 2.5.4 Estimation of Flavonoid content

The aluminum chloride colorimetric technique [10] was modified to estimate flavonoid content. One ml of the extract was mixed with methanol (3 ml), 10 % aluminum chloride (0.2 ml), 1 M potassium acetate (0.2 ml), and distilled water (5.6 ml) at room temperature and incubated for 30 min. The same procedure was performed to create a sample blank, but distilled water was substituted for aluminum chloride. The absorbance was calculated at 420 nm. The standard was Quercetin (1 mg/ml). The flavonoid content was calculated using a standard curve that is represented as Quercetin equivalent (mg/g of the isolated molecule).

#### 2.5.5 Antioxidant activity

In 100 ml of methanol, the 0.0078 g DPPH was dissolved. 1.5 ml of the sample and 1.5 ml of the DPPH solution in methanol were combined in an aliquot (0.2 mM). The samples were incubated at room temperature for 30 min in the dark. At 517 nm, the solution's absorbance was determined. The assay was carried out in the same way as the control, except that methanol was used in place of the sample solution. Analysis was done in triplicate for each extract [26]. The test sample's capacity to scavenge DPPH was assessed by a reduction in absorbance and calculated as shown in Eqn. (7)

(7) $$\text{Antioxidant}\;\text{Activity}\%=\frac{\text{Control}\;\text{absorbance}–\text{extract}\;\text{absorbance}}{\text{Control}\;\text{absorbance}}\times 100$$

### 2.6 Antimicrobial activity

Antimicrobial Activity was evaluated by Agar well diffusion method according to Refs. [27,28]. Muellar Hinton Agar was seeded equally using a sterilized swab from an inoculated solution (saline) containing bacterial strains. For the extra fluid to be absorbed, these plates were left in laminar flow. Wells in seeded agar media were cut using a sterile cork borer. Using a micropipette of 60 μL of extract (1 mg/ml) obtained from different runs was added to plates. Positive control Gentamicin (30 μg/ml) and negative control DMSO (25 μg/ml), as well as negative control (sterile distilled water), was filled uniformly in the wells. The plates were incubated for 24 h at 37 °C. The diameter of the inhibition zone was observed using a caliper in millimeters.

### 2.7 Fourier transforms infrared spectroscopic (FTIR) analysis

FTIR is the most important tool for determining the presence of various chemical bonds and functional groups present in the samples. The distinguishing characteristic of chemical bonds shown in the annotated spectrum is the wavelength of light absorbed. The chemical bond in a substance can be identified by reading the infrared absorption spectra. FTIR analysis was conducted using dried powdered methanol solvent extract from green coconut shells. To create translucent sample discs, (10 mg) of dried crude extract was encapsulated in (100 mg) of KBr pellet. Each extract powder sample was placed in an FTIR spectrophotometer with a scan range of 400–4000 cm^1^ with a resolution of 4 cm^−1^.

### 2.8 Statistical analysis

The findings of each experiment were represented as the mean value ± standard deviation. Using Excel 2010, Design Expert (version 7.0.0), and MATLAB R2019a software the model development, optimization, and other statistical analysis were performed.

## 3 Result and discussion

### 3.1 Response surface methodology analysis

At various levels of independent variables the extraction yield ranged from (19.98–36.1 %), TPC ranged from (7.08–33.46 GAE/gm), TFC ranged from (7.08–33.46 QE/gm), TTC ranged from (70.5–141.09 TAE/gm), and Antioxidant ranged from (66.1–49.98 %) for UAE.

### 3.2 Fitting the model for yield

Based on the BBD, the response of yield for extracts of green coconut shell using UAE was optimized, and the data were fitted to a polynomial equation of second-order. The ANOVA results as shown in Table 1B, were used to check the adequacy and significance of the model. From the results, the significance of the fitted model could be observed at 0.1 % level. The linear coefficients of Sonication time and Solid-solvent ratio were (p < 0.0001) significant at 0.1 % level as shown in the results, whereas, Temperature showed a significant effect at 5 % level. Furthermore, all the factors showed significant quadratic effects on the response. The predictive equation in terms of coded factors is expressed in Equation (8).

(8) $$Y_{Y}=26.4683\;-3.22\;*\;A\;-0.3175\;*\;B+2.9475\;*\;C+1.855\;*\;AB+0.04\;*\;AC+3.995\;*\;BC\;-0.504167\;*A^{2}-1.41917\;*B^{2}+3.65083\;*C^{2}$$

**Table 1B:** ANOVA outcomes for quadratic model of independent variables.

Details of parameters	DF	F-value				
		Yield (%)	Phenols (GAE/gm)	Flavonoids (QE/gm)	Tannins (TAE/gm)	Antioxidant (%)
**Model**	9	283.27*	298.20*	289.39*	403.52*	328.01*
**A-Time**	1	717.86*	436.90*	412.96*	373.33∗	845.17*
**B-Temperature**	1	6.98***	31.94*	30.23*	11.80**	5.53***
**C-Solid-solvent ratio**	1	601.50*	143.63*	145.63*	50.20*	694.94*
**AB**	1	119.12*	394.86*	378.22*	805.10*	130.75*
**AC**	1	0.0554	3.84	3.66	10.33***	0.0642
**BC**	1	552.50*	616.74*	606.41*	540.70*	643.71*
**A**^**2**^	1	9.60***	9.13***	8.44***	139.30*	8.83***
**B**^**2**^	1	76.06*	230.43*	221.49*	1302.45∗	101.70*
**C**^**2**^	1	503.35*	878.37*	857.60*	492.41∗	566.07*

*<0.01; **<0.1; ***<0.05.

From the equation, the negative effects of sonication time and temperature and the positive effect of solid solvent ratio could be observed. Table 1C provides a summary of the results. The model's validity could be confirmed by the non-significant lack of fit value. The R^2^ was 0.9964, which predicted that 99.6 % of the variations could be explained by the fitted model. The adjusted coefficient of determination (R^2^ Adj), which is comparable to R^2^, was 0.9934 and indicated that the observed and predicted values were highly correlated. Additionally, a low coefficient of variation (C.V. = 1.25 %) showed that the experimental results with a great precision level could be trusted and there was little variance in the mean value. Table 1C represents the results of various statistical parameters associated with the fitted model. These findings demonstrated how well the model captured the actual relationship between the independent variables and response.

**Table 1C:** Statistical parameters for quadratic model of independent variables.

Statistical Parameters	Yield (%)	Phenols (GAE/gm)	Flavonoids (QE/gm)	Tannins (TAE/gm)	Antioxidant (%)
**Lack of Fit (F value)**	0.22	0.43	2.40	1.41	0.18
**Pure Error**	0.05	0.20	0.20	1.50	0.05
**Std. Dev.**	0.33	0.54	0.55	1.21	0.31
**Mean**	27.24	20.07	15.08	116.21	57.19
**C.V. %**	1.25	2.71	3.67	1.04	0.55
**R**^**2**^	0.99	0.99	0.99	0.99	0.99
**Adjusted R**^**2**^	0.99	0.99	0.99	0.99	0.99
**Predicted R**^**2**^	0.96	0.97	0.96	0.98	0.96

### 3.3 Fitting the model for phenol

The BBD was used to optimize the response of phenol, and the data from the BBD were fitted to a polynomial equation of second order. The ANOVA results as shown in Table 1B, were used to check the adequacy and significance of the model. From the results, the significance of the fitted model could be observed at 0.1 % level. The linear coefficients of Sonication time, Temperature, and Solid-solvent ratio, all were significant at 0.1 % level as shown in the results. Furthermore, all the factors showed significant quadratic effects (5 % level for the sonication time and 0.1 % levels for the Sonication time and Solid solvent ratio on the response. The predictive equation in terms of coded factors is expressed in Equation (9).

(9) $$Y_{P}=18.0467-4.01575\;*\;A-1.08575\;*\;B+2.3025\;*\;C+5.399\;*\;\text{AB}-0.5325\;*\;\text{AC}+6.7475\;*\;\text{BC}+0.786167\;*A^{2}-3.94883\;*B^{2}+7.70967\;*C^{2}$$

From the equation, the negative effects of sonication time and temperature and the positive effect of solid solvent ratio could be observed. Table 1C provides a summary of the results. The model's validity could be confirmed by the non-significant lack of fit value. The fitted model could account for 99.6 % of the variables, according to the determination coefficient (R^2^) of 0.9970. The Adj R^2^, which is comparable to R^2^, was 0.9937 and indicated that the observed and predicted values were highly correlated. Additionally, a low coefficient of variation (C.V. = 2.71 %) showed that there was little change in the mean value and that the experimental results with a greater precision level could be trusted. Table 1B represents the results of various statistical parameters associated with the fitted model. These findings demonstrated how well the model captured the actual connection between the response and independent factors.

### 3.4 Fitting the model for flavonoids

The BBD was used to optimize the response of flavonoids, and the data from the BBD were fitted to a polynomial equation of second order. The ANOVA results as shown in Table 1B, were used to check the adequacy and significance of the model. From the results, the significance of the fitted model could be observed at 0.1 % level. The linear coefficients of Sonication time, Temperature and Solid-solvent ratio, all were significant at 0.1 % level as shown in the results. Furthermore, all the factors showed significant quadratic effects (5 % level for Sonication time and 0.1 % levels for the Sonication time and Solid solvent ratio on the response. The predictive equation in terms of coded factors is expressed in Equation (10).

(10) $$Y_{F}=13.0433\;-3.9825\;*\;A\;-1.0775\;*\;B+2.365\;*\;C+5.39\;*\;AB\;-0.53\;*\;AC+6.825\;*\;BC+0.770833\;*A^{2}-3.94917\;*B^{2}+7.77083\;*C^{2}$$

From the equation, the negative effects of sonication time and temperature and the positive effect of solid solvent ratio could be observed. Table 1C provides a summary of the results. The model's validity could be confirmed by the non-significant lack of fit value. The fitted model could account for 99.6 % of the variables, according to the determination coefficient (R^2^) of 0.9969. The adjusted determination coefficient (Adj R^2^), which is comparable to R^2^, was 0.9935 and indicated that the observed and predicted values were highly correlated. Additionally, a low coefficient of variation (C.V. = 3.67 %) indicated that the mean value had not changed significantly and that the highly precise experimental results should be believed. Table 1C represents the results of various statistical parameters associated with the fitted model. All of these results showed how accurately the model represented the association between the independent variables and response.

### 3.5 Fitting the model for tannins

Tannin response was optimized using the Box Behnken design, and data from the BBD were fitted to a polynomial equation of second order. The ANOVA results as shown in Table 2, were used to check the adequacy and significance of the model. From the results, the significance of the fitted model could be observed at 0.1 % level. The linear coefficients of Sonication time and Solid-solvent ratio were (p < 0.0001) significant at 0.1 % level as shown in the results, whereas, Temperature showed a significant effect at 1 % level. Furthermore, all the factors showed significant quadratic effects (0.1 % level) on the response. The predictive equation in terms of coded factors is expressed in Equation (11).

(11) $$Y_{T}=122.83\;-8.27\;*\;A\;-1.47\;*\;B+3.0325\;*\;C+17.175\;*\;AB\;-1.945\;*\;AC+14.075\;*\;BC\;-6.84\;*A^{2}-20.915\;*B^{2}+12.86\;*C^{2}$$

**Table 2:** Weight and bias values of the best ANN architecture.

W_ih_	−7.2336	−1.0604	4.7262	W_ho_	2.4338	−19.482	−2.9182	−21.2476	T_h_	4.4098	T_o_	2.2397
	0.43575	2.5957	−2.4158		6.1667	−42.6406	−5.3154	−48.1334		−0.85681		4.4757
	20.2502	−9.4265	−2.8229		6.1947	−42.9296	−5.3605	−48.4433		12.2379		4.5168
	−3.3886	−4.7606	4.7377		9.3231	−51.6052	−5.0571	−60.2125		0.92758		4.3392
					2.5763	−20.0191	−2.9495	−21.9176				2.2433

From the equation, the negative effects of sonication time and temperature and the positive effect of solid solvent ratio could be observed. Table 1C provides a summary of the results. The model's validity could be confirmed by the non-significant lack of fit value. The fitted model could account for 99.78 % of the variables, according to the determination coefficient (R^2^) of 0.9978. The (Adj R^2^), which is comparable to R^2^, was 0.9953 and indicated that the observed and predicted values were highly correlated. Additionally, a low coefficient of variation (C.V. = 1.04 %) suggested that the mean value had not changed considerably and that it was safe to assume the extremely accurate experimental results. Table 1C represents the results of various statistical parameters associated with the fitted model. These outcomes all demonstrated how well the model captured the correlation between the response and independent variables.

### 3.6 Fitting the model for antioxidant

The BBD was used to optimize the antioxidant activity response, and data from the BBD were fitted to a polynomial equation of second order. The ANOVA results as shown in Table 1B, were used to check the adequacy and significance of the model. From the results, the significance of the fitted model could be observed at 0.1 % level. The linear coefficients of Sonication time and Solid-solvent ratio were (p < 0.0001) significant at 0.1 % level as shown in the results, whereas, Temperature showed a significant effect at 5 % level. Furthermore, all the factors showed significant quadratic effects (at 5 % level for Sonication time and at 0.1 % level for Sonication temperature and Solid-solvent ratio) on the response. The predictive equation in terms of coded factors is expressed in Equation (12).

(12) $$Y_{\mathrm{AA}}=56.4683-3.245\;*\;A-0.2625\;*\;B+2.9425\;*\;C+1.805\;*\;AB+0.04\;*\;AC+4.005\;*\;BC-0.449167\;*A^{2}-1.52417\;*B^{2}+3.59583\;*C^{2}$$

From the equation, the negative effects of sonication time and temperature and the positive effect of solid solvent ratio could be observed. Extended exposure to elevated temperatures can result in the deterioration of thermally vulnerable antioxidants. For instance, certain antioxidants, such as vitamin C, are vulnerable to heat and can be annihilated when subjected to extended sonication at high temperatures. Excessive sonication can produce reactive oxygen species (ROS) as a result of cavitation, which is the rapid creation and collapse of bubbles in the liquid. ROS can induce oxidative harm to antioxidants and other substances, hence diminishing their efficacy. The model's validity could be confirmed by the non-significant lack of fit value. The determination coefficient (R^2^) of 0.9973 indicates that the fitted model could explain 99.7 % of the variables. The Adj R^2^, which is equivalent to R^2^, was 0.9943, indicating a strong relationship between the experimental and predicted values. Furthermore, a low coefficient of variation (C.V. = 0.552 %) suggested that the mean value had not changed considerably and that it was safe to assume the extremely accurate experimental results. Table 1C represents the results of various statistical parameters associated with the fitted model. These outcomes all demonstrated how well the model captured the association between the response and independent factors.

### 3.7 Modelling of the extraction process by applying artificial neural network (ANN)

ANN modelling was used to improve the predictive model's accuracy. ‘Tansig’ transfer function and the feed-forward back-propagation method were used to model the experimental data of the extraction process. The input values for the ANN modelling were sonication time, sonication temperature and solid solvent ratio, whereas yield (%), phenols (GAE/gm), flavonoids (QE/gm), tannins (TAE/gm) and antioxidant (%) were the output values. As a result, there were three and five neurons in each of the input and output layers, respectively. Trial and error were used to determine the number of neurons in the hidden layer. The ANN algorithm used 2000 iterations per run, with learning rates ranging from 0.5 to 1. A total of 20,000 NNs were produced after 2000 iterations were performed ten times for each ANN architecture. The constructed MATLAB code was used to fit the experimental data after it had been divided into three equal sections (70 % for training, 15 % for testing, and 15 % for validation, respectively).

**Fig. 1:**
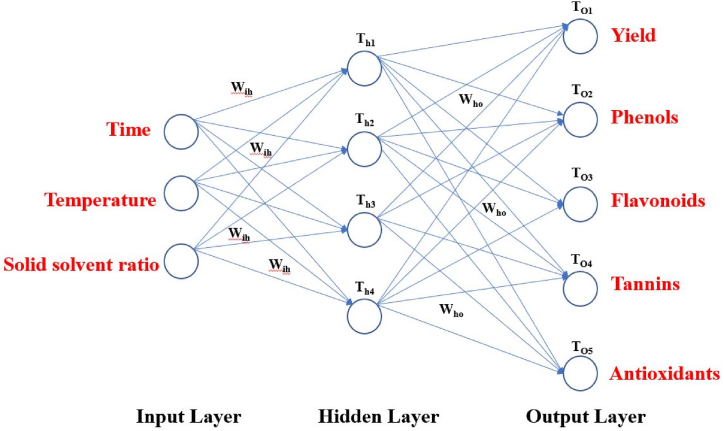
Best ANN Architecture for the mapping of ultrasound assisted extraction from green coconut shell powder. (For interpretation of the references to color in this figure legend, the reader is referred to the Web version of this article.)

The mean square error (MSE) and coefficient of determination (R^2^) were calculated for each run of a certain ANN architecture. The network with the highest R^2^ and lowest MSE values was chosen as the best network. The best ANN architecture for the ultrasound-assisted extraction process was 3 neurons in the input layer, 4 neurons in the hidden layer, and 5 neurons in the output layer (3-4-2). The architecture is shown in Fig. 1. The regression plots during training, testing, and validation of the process are illustrated in Fig. 2A, Fig. 2B, Fig. 2C, Fig. 2D, Fig. 2EA–E. The training state for the initial learning from the data set could be observed in Fig. 2F. The performance plot best epoch and performance value is illustrated in Fig. 2G.

**Fig. 2A:**
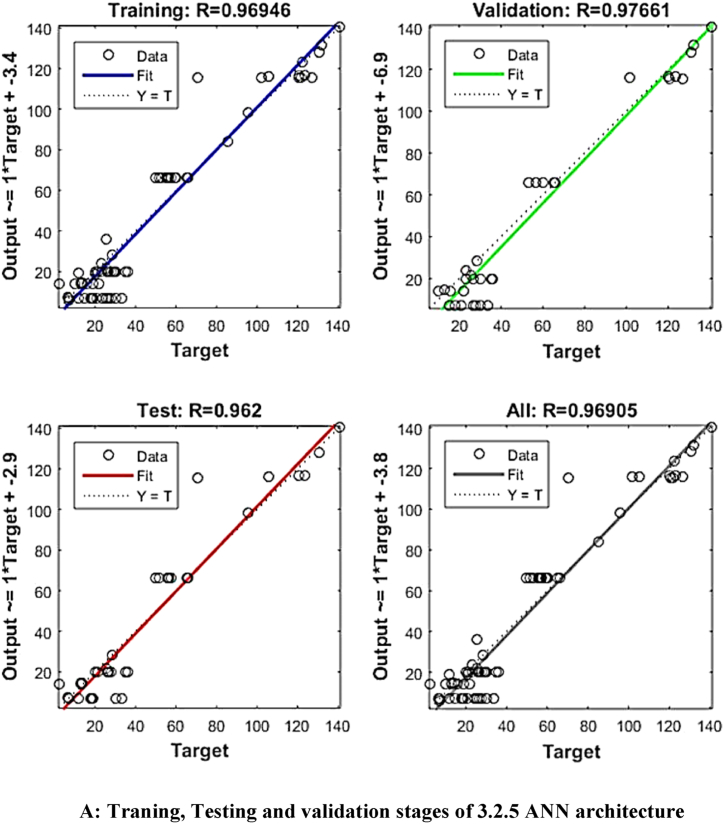
Traning, Testing and validation stages of 3.2.5 ANN architecture.

**Fig. 2B:**
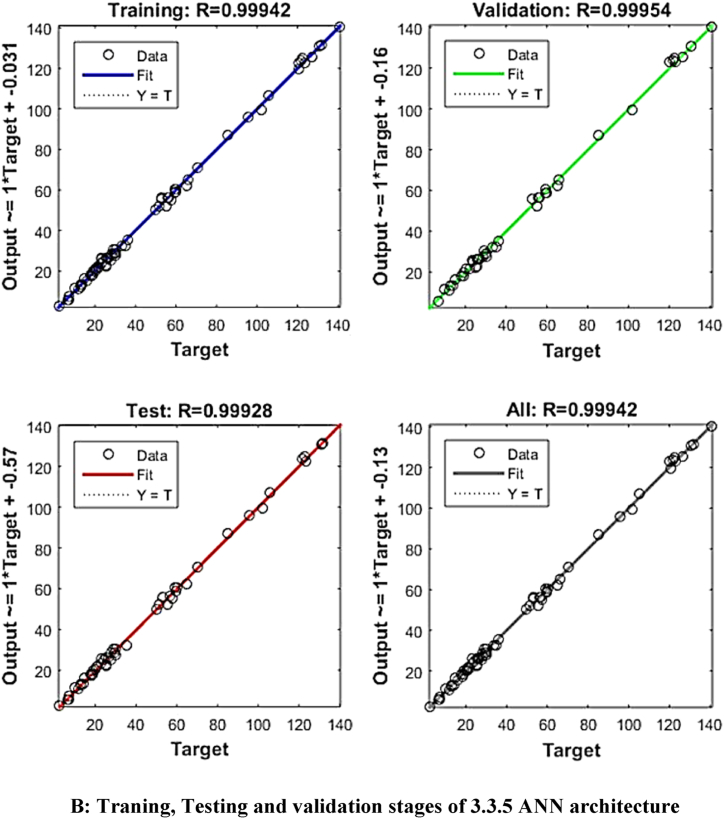
Traning, Testing and validation stages of 3.3.5 ANN architecture.

**Fig. 2C:**
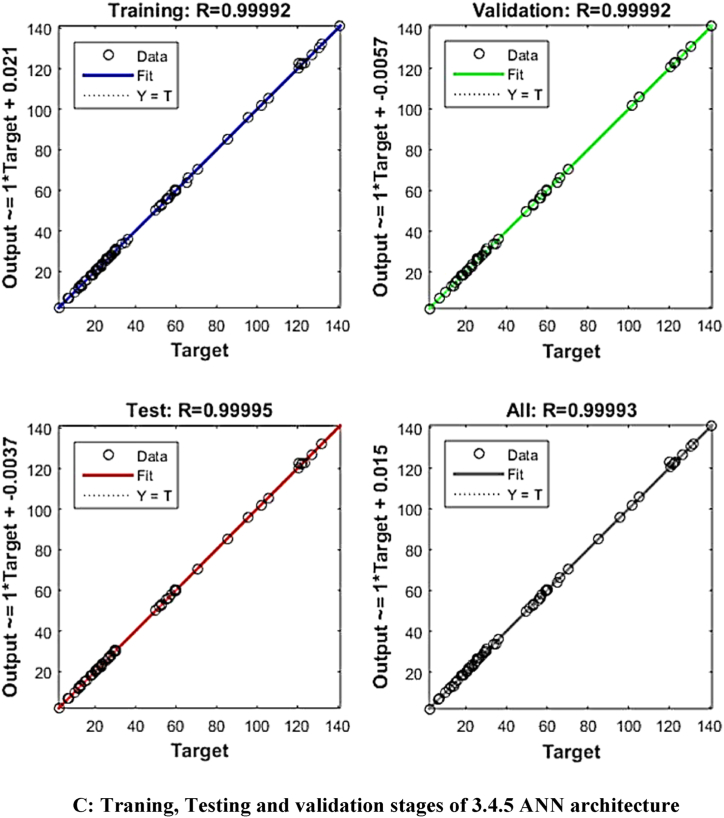
Traning, Testing and validation stages of 3.4.5 ANN architecture.

**Fig. 2D:**
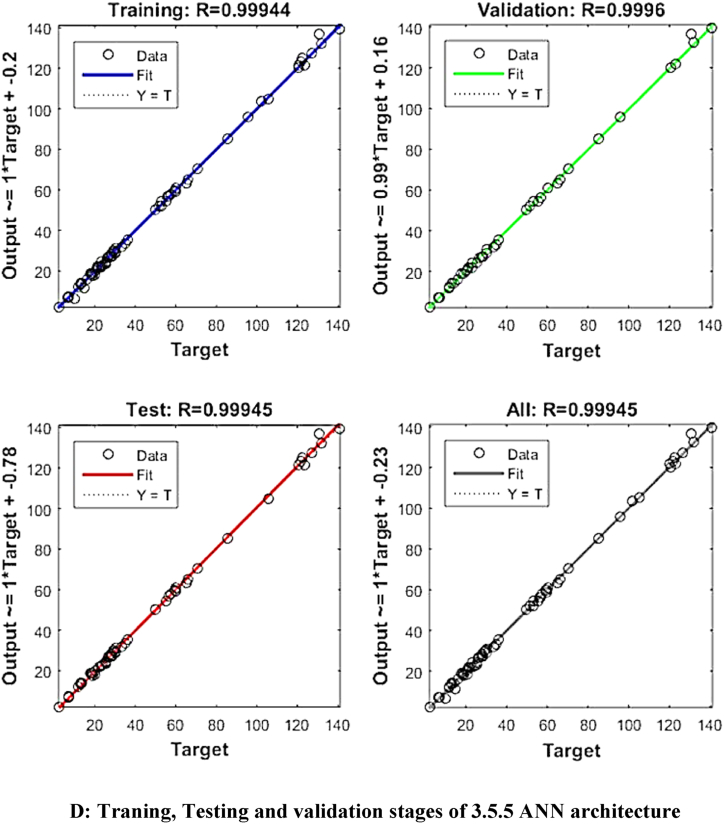
Traning, Testing and validation stages of 3.5.5 ANN architecture.

**Fig. 2E:**
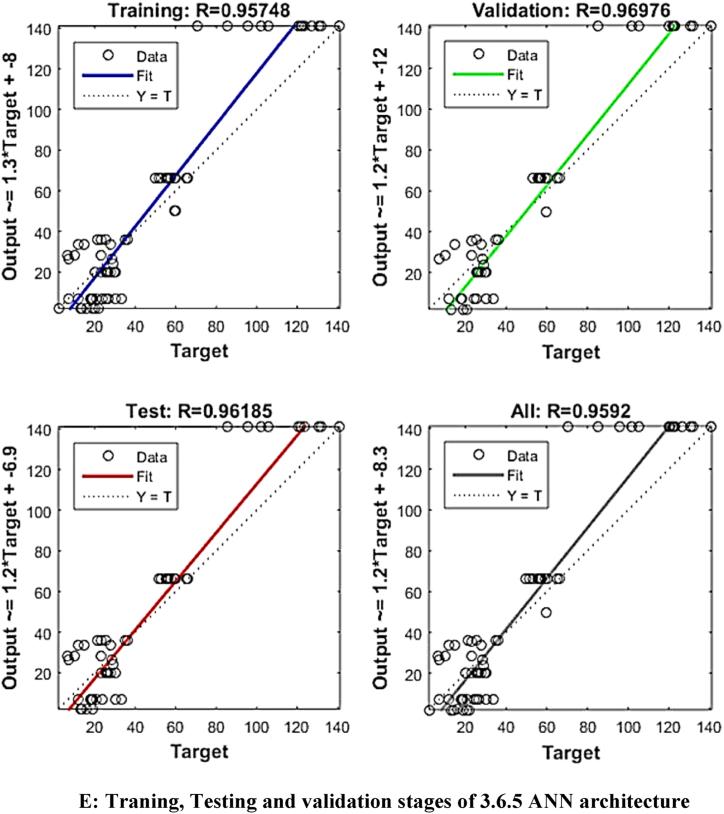
Traning, Testing and validation stages of 3.6.5 ANN architecture.

**Fig. 2F:**
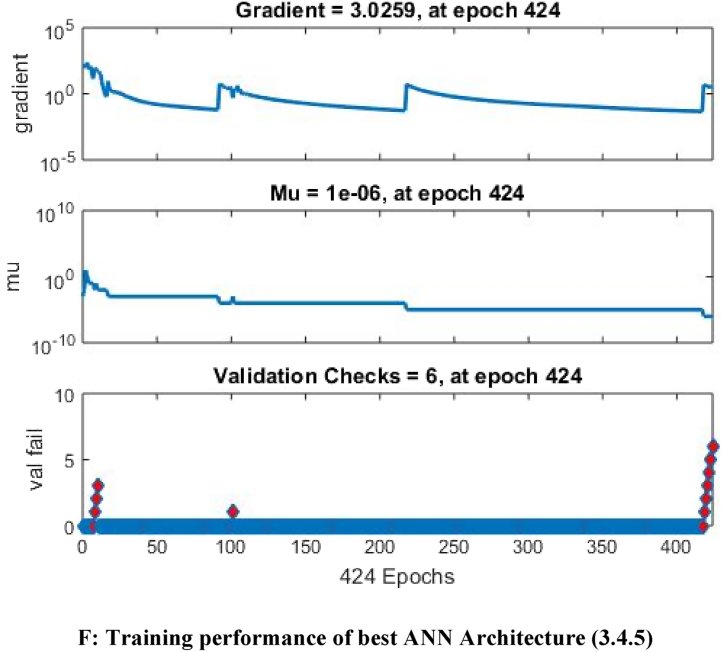
Training performance of best ANN Architecture (3.4.5).

**Fig. 2G:**
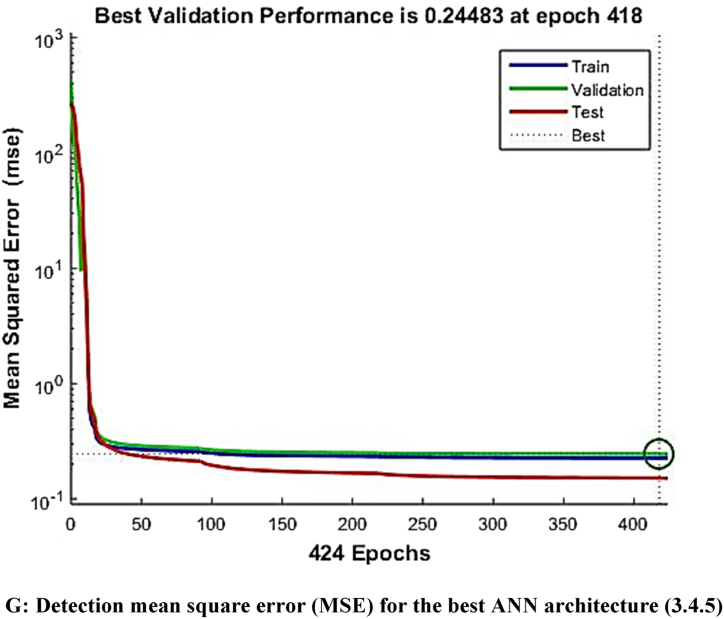
Detection mean square error (MSE) for the best ANN architecture (3.4.5).

The generated code used random number generations to determine the weight and bias values of the best ANN architecture (‘$W_{ih}$’, ‘$W_{ho}$’, ‘$T_{h}$’ and ‘$T_{o}$’). Based on known input, these parameters can be used to calculate the production output of the ultrasound-assisted extraction process viz. Yield (%), Phenols (GAE/g), Flavonoids, (QE/g), Tannins (TAE/g) and Antioxidants (%). Table 2 displays the weight and bias values of the best ANN architecture.

### 3.8 Optimization of process conditions by hybrid ANN-PSO approach

The optimal ANN architecture, 3-4-5 of Time, Temperature, and Solid Solvent Ratio, along with Yield (%), Phenols (GAE/gm), Flavonoids (QE/gm), Tannins (TAE/gm), and Antioxidant (%) was further employed to achieve process parameter optimization. The maximizing of all the responses was accomplished by formulating the fitness function of the optimization process. For the optimization procedure, the modelled parameters as derived from ANN were employed. Consequently, the 3-4-5 ANN architecture's weight and bias values were taken into account as input for the particle swarm optimization (PSO). According to Khawas et al. (2015), hybrid optimization for ANN-GA is a similar notion. The standard particle swarm optimization (SPSO) algorithm was used to calculate PSO. MATLAB R2015a was used to create the SPSO algorithm. The algorithm's starting population size was 40 particles. The weight values varied with the generations from 0.4 to 0.6, whereas the acceleration constants, c_1,_ and c_2_, were both equal to 0.5. 2000 generations were specified as the maximum number of generations. When the iterative process produced the maximum number of generations (2000), it was stopped. The optimum process parameters as determined by hybrid ANN-PSO were time of 15 min, temperature of 33 °C, and solid solvent ratio of 24(w/v). The experimental and predicted values under the optimum process conditions of the extraction process are represented in Table 3.

**Table 3:** Experimental vs. predicted values of the responses under the optimum process conditions.

Type of value	Yield (%)	Phenols (GAE/gm)	Flavonoids (QE/gm)	Tannins (TAE/gm)	Antioxidant (%)
Predicted	38.41	40.99	36.13	176.73	68.39
Experimental	39.32	41.12	37.00	177.32	69.55
% Error	2.31	0.32	2.35	0.33	1.67

### 3.9 Antimicrobial activity

**Fig. 3:**
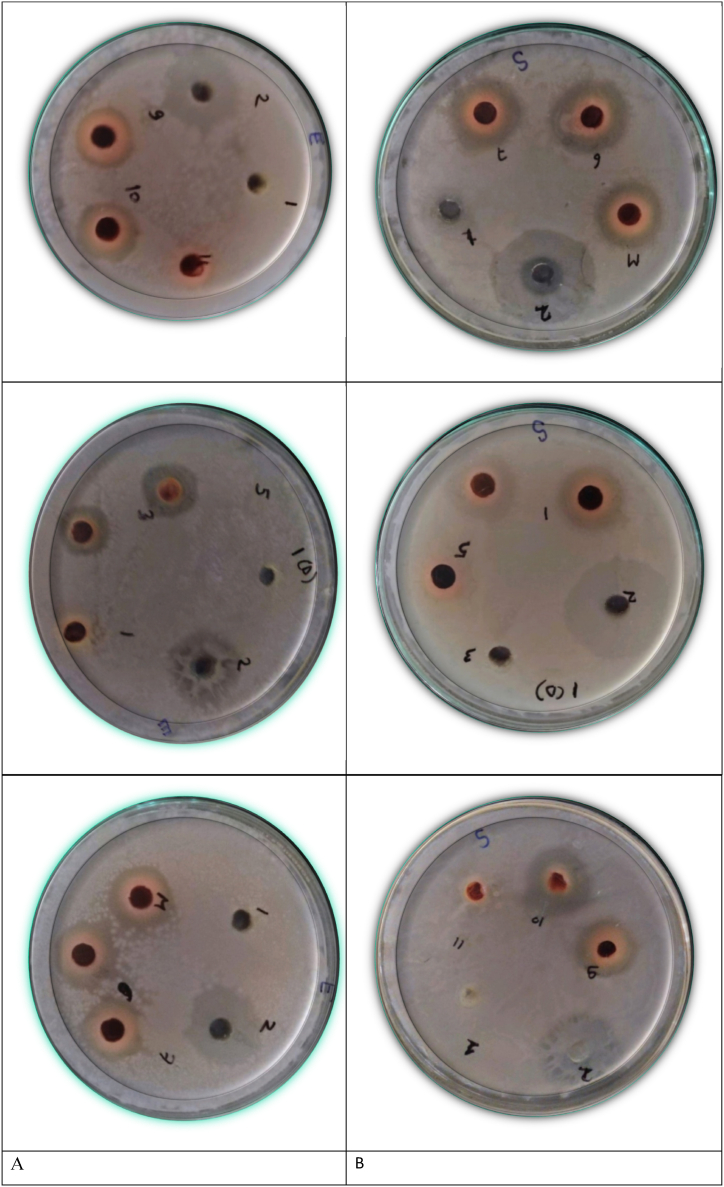
Visual representations of the results of the inhibition zone for (A) *E. coli and (B) S. aureus*.

The antimicrobial activity of extracts was examined based on the diameters of clear inhibition zones surrounding the Petri disks. The visual representations of the results of the inhibition zone are shown in Fig. 3. The maximum inhibition zone for *E. coli* was 9.6 mm and the minimum was 3.7 mm (Fig. 3A) whereas for *S. aureus* the maximum inhibition zone was 11 mm and the minimum was 5.8 mm (Fig. 3B). The previous studies showed the following results [11]. suggested that the antimicrobial activity against *S. aureus* and *E. coli* was 7 mm and 10 mm. According to Rodiah et al.*,* (2018), the Antimicrobial activity of dye extracts was examined based on diameters with clear inhibition zones surrounding the paper disks. If there is no inhibition zone, it is implicit that the dye extract did not possess antimicrobial activity towards the studied bacterial strains. Thus, the antimicrobial effect of the extract was shown when the Ultrasound-assisted extraction technique was employed.

### 3.10 Fourier transform infrared (FT-IR) spectroscopic analysis

**Fig. 4:**
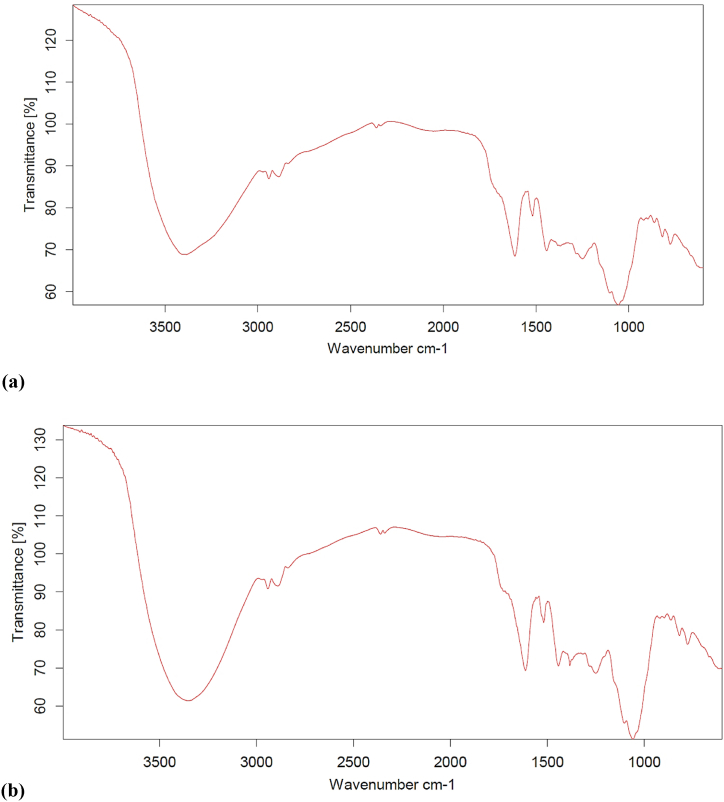
FT-IR spectrum of extract (a) extract obtained from ultrasound; (b) extract obtained from microwave.

Data from the infrared analysis may aid in understanding the sample's chemical composition. An example of evidence for the stretched hydroxyl groups (O–H) in aliphatic and phenolic compounds was the peak of absorption at 3400 cm^−1^. In Ultrasound-assisted extraction, a broad peak at wavenumber 3386.44 cm^−1^ showed O–H bonding with cumulative C–H stretching. A sharp peak at wavenumber 1614.24 cm^−1^ represented C=O bonding. A small peak at wavenumber 1056.99 cm^−1^ depicted C–O bonding whereas in the case of microwave-assisted extract the strong broad peak could be observed at a wavenumber of 3347.81 cm^-^1, showing the stretching of C–H and elongation of O–H bondings. A sharp peak at 1613.20 cm^−1^ represented C=O bond elongation. According to researchers, the prominent peak observed at a wavenumber of 3370 cm^−1^ in the Fourier Transform Infrared (FTIR) spectrum of the extract derived from green coconut shells can be attributed to the stretching vibration of phenolic hydroxyl groups, namely the oxygen-hydrogen (O–H) bond [29,30]; Zheng et al., 2023). Additionally, the broad region surrounding this peak suggests the presence of intermolecular hydrogen bonding among the polyhydroxy aromatic compounds [[31], [32], [33]]. The observed peak at 2940 cm^−1^ can be attributed to the stretching frequencies of the 'C–H′ bonds [34]. A sharp peak at wavenumber 1058.73 cm ^−1^ showed the presence of the C–O group as illustrated in Fig. 4 (a) and (b). These bands were reduced as the number of substituent rings increased, showing cross-linking of the processes occurring between the constituents (tannin and hardener). The peaks in the green coconut shell's FTIR spectrum were indicative of biomass tannins, which had the ideal chemical properties for use as an adhesive compound.

## 4 Conclusions

In this study, an integrated approach of artificial neural network (ANN) and particle swarm optimization was used to standardize the ultrasound-assisted extraction (UAE) process of phytochemicals compounds from the green coconut shell. Results showed the significant effects of Solvent time, temperature, and the solvent-solid ratio on the yield, phenol, tannin, flavonoid content, and antioxidant activity. Three extraction factors (the ratio of solvent to solid, time, and temperature) have also been examined in this study. The optimum process parameters as determined by hybrid ANN-PSO were a Sonication time of 15 min, Temperature of 33 °C, and Solid solvent ratio of 24 (w/v) for a maximum extraction yield of 38.41 %, Total phenol content of 40.99 GAE/g, Total Flavonoid content 36.13 QE/g, Total Tannin content 176.73 TAE/g and Antioxidant activity was 68.39 %. The extract also exhibited antimicrobial activity against *S. aureus* and *E. coli.* Furthermore, this comparative analysis showed that the phytochemicals of green coconut shell extract using MAE were significantly lower than in the UAE. The overall findings of the present investigation demonstrated that UAE was a viable and successful method for extracting Phytochemicals from green coconut shells, as well as indicating the utility of green coconut shells. In popular trash green coconut shells, we quantitively found certain promising phytochemicals. Every year, imports of phytochemicals, which are all necessary raw materials for the food and pharmaceutical industries, are made. We can save a sizable sum of foreign currency if we can produce phytochemicals using green coconut shells. Annually, people worldwide dispose of one million metric tons of green coconut shells. As a result, its significance will increase in relation to concerns about environmental pollution. Hence, the implementation of a strategy to establish an interconnected sector focused on processing waste materials from plants and extracting extremely valuable phytochemicals, which are crucial for the food and pharmaceutical industries, would provide significant benefits.

## Data availability

No data was used for the research described in the article.

## Funding

Project No. TKP2021-NKTA-32 has been implemented with support from the 10.13039/501100012550National Research, Development, and Innovation Fund of Hungary, financed under the TKP2021-NKTA funding scheme.

## CRediT authorship contribution statement

**Poornima Singh:** Writing – original draft, Software, Methodology, Investigation, Formal analysis, Data curation. **Vinay Kumar Pandey:** Writing – original draft, Software, Methodology, Investigation, Formal analysis, Data curation. **Sourav Chakraborty:** Writing – original draft, Validation, Methodology, Investigation, Formal analysis, Data curation. **Kshirod Kumar Dash:** Writing – review & editing, Writing – original draft, Supervision, Resources, Project administration, Funding acquisition, Formal analysis, Data curation, Conceptualization. **Rahul Singh:** Writing – review & editing, Writing – original draft, Validation, Supervision, Resources, Investigation, Funding acquisition, Formal analysis, Data curation, Conceptualization. **Ayaz Mukarram shaikh:** Writing – review & editing, Validation, Software, Methodology, Investigation, Data curation. **Kovács Béla:** Writing – review & editing, Resources, Project administration, Funding acquisition, Formal analysis, Data curation.

## Declaration of competing interest

The authors declare that they have no known competing financial interests or personal relationships that could have appeared to influence the work reported in this paper.
